# Exploring the role of insulin resistance in bridging the metabolic syndrome and Alzheimer’s disease-a review of mechanistic studies

**DOI:** 10.3389/fendo.2025.1614006

**Published:** 2025-10-08

**Authors:** Shilei Wang, Yuqing Shi, Rui Xin, Hailan Kang, Huazhong Xiong, Jixiang Ren

**Affiliations:** ^1^ College of Traditional Chinese Medicine, Changchun University of Chinese Medicine, Changchun, Jilin, China; ^2^ Affiliated Hospital to Changchun University of Chinese Medicine, Changchun, Jilin, China

**Keywords:** metabolic syndrome, insulin resistance, cognitive impairment, Alzheimer’s disease, mechanism research

## Abstract

The association between metabolic syndrome (MetS) and Alzheimer’s disease (AD) has attracted widespread attention; nevertheless, the precise mechanism of action between the two is not yet fully elucidated. This review systematically explores the complex mechanisms of insulin resistance (IR) in MetS and AD. We first detail the intrinsic mechanisms of insulin resistance and emphasize its central role in the pathophysiology of MetS. Further, we reveal the underlying mechanisms by which insulin resistance in turn triggers AD through a multidimensional pathway that promotes the accumulation of pathological products, induces blood-brain barrier dysfunction, impairs neuroplasticity, induces neuroinflammatory responses, aberrantly activates the renin-angiotensin-aldosterone system, and exacerbates oxidative stress. In addition, we summarize potential strategies for targeting IR in AD treatment and demonstrate the promising prospects for improving insulin resistance in promoting cognitive recovery. This study offers a novel theoretical framework for elucidating the intricate relationship between MetS and AD. Furthermore, it provides a scientific foundation for the formulation of preventive and therapeutic strategies for metabolic and neurodegenerative diseases.

## 1 Introduction

Insulin resistance (IR), as a pathological state in which the body’s response to the physiological effects of insulin is reduced, has become a central issue in the field of modern metabolic diseases (1, 2). This metabolic disorder not only impairs the core physiological function of insulin in regulating glucose uptake and utilization but also extensively interferes with key metabolic pathways such as lipid metabolism and protein homeostasis, thus providing a common pathophysiological basis for numerous metabolic diseases (3).

With the acceleration of urbanization, the prevalence of sedentary behavior, and the global spread of high-calorie dietary patterns, the prevalence of MetS has continued to increase, and it has become a major challenge that threatens the security of global public health (4). Epidemiological research indicates a prevalence rate of MetS at 41.8% within the United States, while in China, the figure stands at 33.9% (5, 6), highlighting its widespread prevalence. MetS is a clinical syndrome with IR as the core pathogenesis, characterized by central obesity, elevated blood pressure, abnormal fasting glucose, and lipid metabolism disorders (7, 8). These metabolic abnormalities significantly increase the risk of cardiovascular disease (9–11).

With the acceleration of global population aging, the incidence of dementia, represented by Alzheimer’s disease (AD), has shown exponential growth. Epidemiological research reveals that presently, over 55 million individuals are afflicted with dementia globally, with projections anticipating that this figure will surpass 130 million by the year 2050 (https://www.who.int/zh). AD, as a major subtype of dementia, accounts for about 60-70% of all cases, and its progressive neurodegenerative lesions not only pose a serious threat to the quality of life of the elderly population but also become a significant burden to the public health system (12). However, the overall efficacy of current clinical therapeutic regimens is still unsatisfactory (13), making the identification and characterization of AD-related risk factors a priority for prevention and control strategies - targeting and modifying interventional risk factors, may provide a key target for intervention in the disease process. Notably, prior research have concentrated on the impact of an individual metabolic factor on AD, ignoring the fact that different metabolic factors might collectively or synergistically contribute to the heightened likelihood of developing AD (14). Therefore, studies targeting the MetS as a group of risk factors may be more helpful in the prevention and management of AD.

Accumulating epidemiological and clinical research evidence shows that MetS contributes significantly to the progression of AD (15–17). Particularly in older age groups, patients with MetS are more likely to develop AD, and this trend is more pronounced in women (18). The close relationship between these two diseases is centered on the fulcrum of IR (19), which is not only a core pathological mechanism of MetS, but also closely related to the pathological process of AD (20, 21). Therefore, this review aims to thoroughly analyze the multidimensional mechanism of IR as a key pathological hub in the association between MetS and AD. By integrating multidisciplinary evidence from molecular biology, neuropathology, and clinical medicine, the cascade response network between IR-MetS-AD will be systematically elucidated, and potential preventive and therapeutic strategies will be explored.

## 2 Methods

### 2.1 Literature search criteria

Databases: A systematic search was conducted in PubMed, Web of Science, and Scopus.

Search Terms:

PubMed: (“Insulin Resistance”[MeSH] OR insulin resistance) AND (“Metabolic Syndrome”[MeSH] OR metabolic syndrome) AND (“Alzheimer’s disease”[MeSH] OR Alzheimer’s disease) AND (“Cognitive impairment”[MeSH] OR cognitive impairment) AND (mechanism OR pathogenesis OR molecular pathway).
Web of Science: TS=(insulin resistance) AND TS=(metabolic syndrome) AND TS=(Alzheimer’s disease) AND TS=(mechanism OR pathogenesis).
Scopus: TITLE-ABS-KEY(insulin resistance) AND TITLE-ABS-KEY(metabolic syndrome) AND TITLE-ABS-KEY(Alzheimer disease) AND TITLE-ABS-KEY(cognitive impairment OR mild cognitive impairment OR cognitive dysfunction) AND TITLE-ABS-KEY(mechanism).
Language/Time: English-language studies published up to August 2025 were prioritized.

### 2.2 Inclusion/exclusion criteria

Inclusion criteria:

Original research articles, reviews, or meta-analyses that explore the role of insulin resistance (IR) as a bridge between metabolic syndrome (MetS) and Alzheimer’s disease (AD).
Studies involving IR and its relationship with cognitive function, neurodegeneration, or AD pathological mechanisms (e.g., Aβ deposition, Tau phosphorylation, blood-brain barrier dysfunction, RAAS system activation, oxidative stress, neuroinflammation, etc.).

Exclusion criteria:

Studies based on non-mammalian models.
Case reports, conference abstracts, and studies lacking full-text availability or complete data.

## 3 Pathophysiologic mechanisms of insulin resistance

### 3.1 Insulin signaling

**Figure 1:**
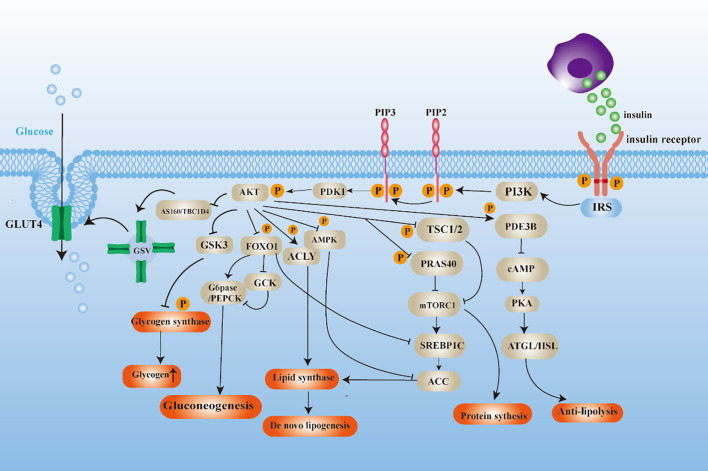
Diagram of insulin signaling mechanism.

The binding of insulin to the insulin receptor initiates a sequence of subsequent reactions, including the recruitment and phosphorylation of various proteins. The composition of these proteins is primarily constituted by IRS, PI3K, and AKT subtypes, which serve as the initiating agents for a series of insulin responses (22). The activation of AKT manifests diverse characteristics that result in varied distal signaling responses to insulin in target tissues (**Figure 1**). (a) In the complex metabolic regulatory network, AKT substrates include glycogen synthase kinase-3(GSK3), and phosphorylation-inactivation of GSK3 is a pivotal junction in the glycogen synthesis (23). Additionally, the transcription factor Forkhead Box O1 (FOXO1) undergoes nucleoplasmic shuttling facilitated by AKT kinase phosphorylation, leading to inhibition of its transcriptional activity, which represses the expression of glycoheterotrophic genes (24, 25). (b) Tuberous Sclerosis Complex 1/2 (TSC1/2) with Proline-Rich Akt Substrate 40 (PRAS40) acts as a negative regulator of the mTORC1 signaling pathway, modulating protein translation initiation and lipid biosynthesis pathways by inhibiting mTORC1 activity (26, 27). (c) Dephosphorylation of acetyl-CoA carboxylase (ACC) and activation of ATP citrate lyase (ACLY) phosphorylation lead to a constitutive increase in *de novo* lipogenesis (DNL) (28, 29). (d) Phosphodiesterase 3B (PDE3B) maintains lipid homeostasis and participates in the negative regulation of adipocyte lipolysis by inhibiting adipose triglyceride lipase (ATGL) and hormone-sensitive lipase (HSL) activities (30, 31).

In summary, insulin plays a pivotal role in the management of glucose and lipid balance. Following a meal, insulin secreted by pancreatic β-cells instigates anabolic programs while impeding catabolic pathways. In glucose metabolism, insulin stimulates glucose uptake in skeletal muscle, hepar, fatty tissue and other tissues, and accelerates glycogen and lipid synthesis. Furthermore, insulin effectively regulates glucose output from the liver by down-regulating gluconeogenic gene expression and inhibiting lipolysis, thereby maintaining energy balance in the body.

### 3.2 Mechanisms of insulin resistance

Insulin resistance is a complex pathophysiological process rooted in genetic susceptibility, initiated by core metabolic disturbances, and progressively amplified and sustained through cellular stress and systemic inflammation. Its underlying mechanisms can be systematically elaborated from the following three interrelated levels.

#### 3.2.1 Core drivers: genetic susceptibility and ectopic lipid deposition

The development of insulin resistance (IR) is fundamentally grounded in genetic susceptibility. Clinical data from 220 Caucasian and 36 African American children and their parents revealed that children with at least one IR-affected parent exhibited significantly elevated insulin levels (32). Genetic background influences insulin sensitivity through multiple pathways, including: monogenic mutations (e.g., in AKT2 and INSR genes) that lead to severe insulin resistance phenotypes (33, 34); polygenic inheritance resulting from the cumulative small effects of multiple genes, such as PLA2G6 (which enhances sensitivity) and VGLL3 (which reduces sensitivity), identified through genome-wide association studies (35); and epigenetic modifications (e.g., DNA methylation) that regulate gene expression without altering the DNA sequence (36, 37).

Against this backdrop of genetic susceptibility, ectopic lipid deposition represents the most critical metabolic driver of insulin resistance. Although obesity is a well-established risk factor for type 2 diabetes mellitus (T2DM) (38), the crux of its pathophysiology lies in the abnormal accumulation of lipids in non-adipose tissues such as the liver and skeletal muscle (39–41). Studies have demonstrated a significant positive correlation between intrahepatic lipid content and the insulin resistance index (HOMA-IR), while weight loss interventions that reduce hepatic fat can effectively reverse hepatic insulin resistance and improve hyperglycemia (42). This lipotoxicity directly interferes with insulin signaling pathways, thereby constituting the initiating event in the development of insulin resistance.

#### 3.2.2 Key nexus: cellular stress and dysfunction

Core metabolic disturbances triggered by ectopic lipid deposition and nutrient excess further activate cellular stress responses. These responses act as critical pathological hubs, closely linking upstream metabolic signals to downstream impairment of insulin signaling.

Mitochondrial Dysfunction and Oxidative Stress: Mitochondria are the primary source of superoxide and hydrogen peroxide (43). Excessive mitochondrial ROS production promotes a pro-oxidative shift in redox homeostasis, leading to disrupted redox signaling and oxidative damage, collectively termed oxidative stress (44), which contributes to the pathogenesis of insulin resistance and diabetes (45). A cross-sectional study involving overweight, obese, and normal-weight individuals demonstrated that oxidative stress levels were significantly positively correlated with the degree of insulin resistance, particularly in overweight and obese subjects. Specifically, oxidative stress markers such as total oxidant capacity (TOC) were positively correlated with the insulin resistance index (HOMA-IR), whereas total antioxidant capacity (TAC) showed a negative correlation with HOMA-IR (46).

Endoplasmic Reticulum Stress: The native structure of insulin is formed within the endoplasmic reticulum (ER); thus, proper maintenance of ER homeostasis is essential for the metabolic stability and function of pancreatic β-cells (47, 48). Studies using cell cultures and mouse models have revealed that obesity induces ER stress, which suppresses insulin receptor signaling through the overactivation of c-Jun N-terminal kinase (JNK) (49). Clinical research has further shown that serum ER stress markers are significantly elevated in patients with type 2 diabetes mellitus (T2DM) compared to healthy individuals, and their levels are positively correlated with the insulin resistance index (HOMA-IR) (50).

#### 3.2.3 Systemic amplification: environmental factors and metaflammation

Cellular stress and dysfunction are not isolated events; rather, under the synergistic influence of adverse environmental factors, they are amplified into systemic metaflammation, ultimately forming a self-reinforcing vicious cycle.

##### 3.2.3.1 Synergistic effects of environmental factors

Multiple environmental exposures can significantly exacerbate insulin resistance. For instance, Western dietary patterns—characterized by high consumption of red meat, sugar, and saturated fats—directly promote ectopic lipid deposition and inflammatory responses. In contrast, healthier dietary patterns such as the Mediterranean diet have demonstrated clear protective effects (42, 51, 52). Additionally, air pollution, including long-term exposure to particulate matter (e.g., PM2.5, PM10), is closely associated with the onset and progression of insulin resistance, with its adverse effects being partially mitigated by physical activity (53–55). Concurrently, stressors in the home, workplace, and community are linked to accelerated aging and alterations in metabolic and immune function (56). For example, a cohort study demonstrated an independent association between occupational stress and insulin resistance (57). Similarly, animal studies have revealed that stress increases both insulin secretion and insulin resistance (58).

##### 3.2.3.2 Metaflammation

Metaflammation represents the core mechanism through which insulin resistance evolves from localized cellular damage into a systemic disease. Nutrient excess and adipocyte hypertrophy recruit and activate immune cells—such as macrophages and T cells—to infiltrate adipose tissue, where they secrete large quantities of pro-inflammatory cytokines (e.g., TNF-α, IL-1β, IL-6) (59, 60). This chronic, low-grade inflammatory state accelerates the spillover of lipids from adipose tissue into skeletal muscle and liver, leading to ectopic lipid deposition and insulin resistance in these tissues (61). Consequently, metaflammation acts not only as a downstream consequence of cellular stress but also as a key upstream driver that perpetuates and amplifies insulin resistance, establishing a difficult-to-break vicious cycle.

## 4 Insulin resistance and metabolic syndrome

The onset and progression of MetS is a multifactorial and complex process, in which IR plays a central role (3, 62), and there is a close correlation with other components of MetS.

### 4.1 Obesity

A vicious cycle has been established between IR and obesity, which are reciprocally causal. IR and its concomitant hyperinsulinemia trigger excessive fat storage, imbalanced energy metabolism, and disrupted neuroendocrine regulation. These factors act synergistically to promote the development and progression of obesity (63–65). A clinical study lends empirical support to this theory, demonstrating that specific, partial inhibition of insulin production is effective in alleviating high-fat diet-induced obesity (66). However, obesity itself has been shown to exacerbate IR (59), in which visceral adipose tissue plays a central role in the induction of IR (67), thereby consolidating a metabolic disorder that is difficult to reverse.

### 4.2 Hyperglycemia

Insulin acts a crucial regulatory part in the preservation of glucose homeostasis, including glucose uptake, gluconeogenesis and other physiological processes (68–70). However, in states of IR, the capacity of insulin to regulate blood glucose is markedly compromised (71). Specifically, impairment of the insulin signaling pathway hinders the translocation of glucose transporter proteins (e.g., GLUT4) to the cell membrane, which in turn reduces the efficiency of cellular glucose uptake (72). Furthermore, the liver’s increased production and secretion of glucose, prompted by IR, contributes to the exacerbation of blood glucose levels (73).

### 4.3 Abnormalities of lipid metabolism

In a state of IR, adipose tissue becomes less sensitive to insulin’s anti-lipolytic action, which consequently results in a heightened secretion of free fatty acids (FFA) (74). This increased influx of FFA into the liver and muscle tissue promotes triglyceride synthesis (75). A study conducted on non-obese men revealed that individuals with low insulin sensitivity and low lipocalin levels exhibited an elevated risk of developing fatty liver disease and dyslipidemia (76). A separate study further corroborates the notion that hepatic IR alone is sufficient to induce dyslipidemia, thereby accelerating the onset of atherosclerosis associated with the MetS (77). Conversely, abnormalities in lipid metabolism, especially the accumulation of intracellular triglycerides, activate novel protein kinase C, which impairs insulin signaling and has been implicated as one of the key mechanisms of IR (78).

### 4.4 Hypertension

IR impacts glucose and lipid metabolism, as well as the RAAS, which contributes to elevated vascular tension (79). A cross-sectional analysis indicated that Participants with prehypertension tend to have higher insulin levels and more significant IR compared to healthy individuals (80). In such cases, compensatory hyperinsulinemia in insulin-resistant individuals has been observed to intensify salt reabsorption in the renal tubules, leading to salt excess and increased vascular tension (81). Furthermore, excessive activation of RAAS has been demonstrated to exert inhibitory effects on insulin signaling, which may further exacerbate IR, thereby creating a vicious cycle (82).

In summary, IR occupies a central position in the pathophysiologic mechanisms of MetS. It exerts a substantial influence on the development of MetS components by broadly impacting numerous metabolic pathways and physiological processes. Moreover, it functions as a pivotal factor in perpetuating the metabolic disordered state. Consequently, the development of intervention strategies targeting IR is of particular importance, not only for the prevention of MetS but also for the treatment of MetS and its related diseases.

## 5 Association of metabolic syndrome with Alzheimer’s disease

### 5.1 Impact of metabolic syndrome on cognitive functioning

Several evidence indicates that metabolic syndrome (MetS) exerts a significant adverse impact on cognitive function. At the population level, a systematic review and dose-response meta-analysis of 30 studies provides robust support: 17 of these studies explicitly report that the presence of MetS accelerates cognitive decline, although a minority (2 studies) present opposing findings (83). A larger neuroimaging study involving over 37,000 participants further elucidates the potential neuropathological underpinnings, revealing significant associations between MetS and reduced brain volume, increased cerebrovascular pathology, and poorer performance across multiple cognitive domains (84). At the mechanistic level, animal model studies offer direct biological evidence. Research demonstrates that diet-induced MetS in animals not only leads to glucolipid metabolic disturbances but also results in significant impairments in learning and memory, accompanied by pathological alterations in the hippocampus (85, 86).

### 5.2 Epidemiologic studies of metabolic syndrome and Alzheimer’s disease risk

Multiple large-scale population studies have consistently demonstrated that metabolic syndrome and its individual components are significantly associated with an increased risk of dementia. Analyzing in depth a large dataset covering 466,788 individuals, the results of the study revealed a strong association between the MetS and its components with a markedly higher risk of dementia. Specifically, individuals with MetS faced a 25% increased risk of all-cause dementia, with a significant 50% increased risk of vascular dementia (VD) (87). In addition, a long-term follow-up study of nearly 200,000 elderly participants without dementia further confirmed that MetS was linked to a 12% elevated risk of dementia onset, and this risk association showed a more pronounced trend with longer follow-up (88). Another analysis based on large-scale health data from people aged 50 to 69 years in Korea also found that MetS and its early states were remarkably connected with an enhanced incidence of AD, with a 20% higher risk of developing AD in the early MetS group compared with the non-MetS group, and a further increase in risk to 39% in the MetS group (89). Collectively, these findings underscore the complexity of metabolic syndrome (MetS) as a risk factor for dementia. Not only does MetS independently influence Alzheimer’s disease (AD) and vascular dementia (VaD), but it may also play a central role in mixed dementia, where these two pathologies intersect. This critical area warrants further in-depth investigation in future research.

### 5.3 Direct and indirect associations between components of the metabolic syndrome and Alzheimer’s disease

#### 5.3.1 Obesity and Alzheimer’s disease

Studies have shown that obesity in midlife increases the risk of developing AD independently of other factors. However, in the later stages of life, especially those obese individuals who are metabolically healthy may exhibit some protection against AD pathology (90). Obesity-induced chronic peripheral inflammation is not limited to other parts of the body, but may also spread to the brain, triggering a neuroinflammatory response, which in turn accelerates the course of cognitive impairment (91–93). In addition, obesity-induced leptin resistance further exacerbates Cognitive impairment and the advancement of AD (94). Nonetheless, existing studies present some complexity: some evidence suggests that obesity in midlife may play a role as a protective factor for AD, whereas midlife underweight status might contribute to an increased risk of dementia (95).

#### 5.3.2 Hyperglycemia and Alzheimer’s disease

Cognitive dysfunction is increasingly being recognized by the academic community as an important comorbidity of diabetes (96). A long-term cohort analysis, upon correction for potential confounders like age, sex, and educational attainment, disclosed that diabetic patients had a 65% higher risk of developing AD compared to the non-diabetic group (97). Another cohort study further confirmed that the risk ratio (HR) for AD was significantly higher in diabetic patients, especially in the group of older diabetic women (98). In addition, the drugs traditionally used to treat type 2 diabetes showed activity in improving the cognitive health of patients with AD, which also confirmed the similarity of the pathogenesis of these two diseases (99). In recent years, with the advancing depth of research, there is more and more evidence that AD may be a brain-specific type of diabetes mellitus, the so-called “type 3 diabetes mellitus” (100, 101).

#### 5.3.3 Dyslipidemia and Alzheimer’s disease

The connection between triglycerides and AD risk may be modulated by age, showing a complex nonlinear pattern. Specifically, higher triglyceride levels were markedly associated with an added risk of dementia in people under 60 years of age (102). However, in older populations, this relationship is reversed, and a reduced likelihood of AD has been associated with high triglyceride levels (102, 103). Furthermore, HDL can reduce the risk of AD due to its vasculoprotective function, which is mediated by mechanisms including increased Aβ clearance and induction of endothelial nitric oxide production (104). On the other hand, elevated cholesterol levels are regarded as a possible risk factor for the onset of AD (105). Cholesterol levels are generally higher in patients with AD compared to healthy individuals and are strongly associated with the accumulation of phosphorylated tau in the brain (106). Notably, an 8-year cohort study revealed an association between sustained statin use and a significantly lower risk of incident AD (107). However, there is also strong evidence that statin use is not effective in preventing memory disorders in the elderly at risk for vascular disease (108).

#### 5.3.4 Hypertension and Alzheimer’s disease

Hypertension shows a positive correlation with neuropathological changes in Alzheimer’s disease, which is closely associated with an increase in plaques and neurofibrillary tangles in the brain, and has emerged as a significant contributing factor for the disease (109). Notably, hypertensive patients in the middle age period face a higher risk of cognitive impairment compared to the elderly population (110). Specifically, midlife stage 1 and stage 2 systolic hypertension are associated with an increased risk of AD of 18% and 25%, respectively (111). In response to this situation, the use of antihypertensive drugs is particularly important. Pertinent research has indicated that antihypertensive medications are efficacious in lowering the risk of AD among hypertensive individuals. To illustrate, a meta-analysis in a prospective cohort study revealed that compared with hypertensive people who did not use antihypertensive medication, those who regularly used antihypertensive medication had a 12% lower risk of dementia and a 16% lower risk of AD (112).

Overall, there is a clear link between components of the MetS and AD, with hyperglycemia having a particularly strong effect on AD. However, when assessing the impact of the MetS on AD risk, It is vital to take full account of the impact of age. In particular, when exploring the relationship between obesity dyslipidemia and AD, the influence of the age factor is particularly important and should not be ignored.

## 6 Insulin resistance and Alzheimer’s disease

### 6.1 Sources of insulin in the brain

Insulin levels in cerebrospinal fluid (CSF) are significantly lower than those in plasma, yet a strong correlation exists between the two, suggesting that brain insulin primarily originates from circulating pancreatic insulin (113). Insulin enters the central nervous system (CNS) via selective, saturable transport mechanisms across the blood-brain barrier (BBB) capillary endothelial cells (114, 115). Additionally, the choroid plexus—a key structure in CSF production—has been identified as another important source of insulin within the CNS (116). Recent studies have further detected insulin expression in the dorsal vagal complex (DVC) of the hindbrain (117), lending additional support to the possibility of local insulin synthesis in the brain.

### 6.2 Definition of brain insulin resistance

Brain insulin resistance is characterized by a diminished response to insulin signaling within the central nervous system (CNS), primarily involving neurons and/or glial cells. The underlying mechanisms include downregulation of insulin receptors, impaired insulin-receptor binding, or defective activation of insulin signaling cascades. At the cellular level, this dysfunction may manifest as impaired neuroplasticity, altered receptor modulation, or disrupted neurotransmitter release in neurons. Alternatively, it may directly impair insulin-dependent metabolic processes, such as glucose uptake or glycogen synthesis. Functionally, brain insulin resistance can present as dysregulation of central brain energy metabolism and peripheral glucolipid metabolism, or deficits in cognitive and emotional functions (118, 119).

### 6.3 Role of insulin signaling pathways in brain function

In the complex network of insulin signaling, phosphatidylinositol 3-kinase (PI3K) and protein kinase B (Akt) play indispensable roles as key kinases. They not only regulate neuronal plasticity and survival but also participate in neurotransmitter transport, which is essential for maintaining normal neural function (120, 121). In particular, brain areas closely connected with cognitive functions, such as the hippocampus, highly express insulin receptors, underscoring the importance of insulin in these regions (122, 123). The PI3K/Akt sequential response further influences various subsequent pathways, among them mTORC1, GSK3β, and the FoxO group of transcription factors, which are pivotal in in basic brain function (124). For instance, protein synthesis orchestrated by mTORC1 is pivotal for neural adaptability and the regulation of autophagic processes; however, misregulation of this process can precipitate neuronal apoptosis and the inception of neurodegenerative disorders (125–127). Furthermore, GSK3β is instrumental in governing various facets of neuronal activity, including neurogenesis and synaptic function (128, 129), and its ability to phosphorylate tau proteins and increase the amount of β-amyloid has been closely linked to the pathogenesis of AD (130, 131). FOXO3, a crucial component of the FOXO gene family, is extensively present in various organs and tissues of the human body. Changes in its protein expression level and post-translational modification status is crucial for upholding the stability of the body’s internal milieu and mitigating aging-related pathologies (132). In addition, insulin activates the MAPK cascade response, which regulates cell proliferation, differentiation, and apoptosis, and contributes to the maintenance of normal neuronal cell function and synaptic plasticity (133, 134). Collectively, these investigations indicate a profound and intricate association between the insulin signaling mechanism and the functionality of neurons and synapses. In summary, as described in the literature, insulin signaling is widely distributed throughout the brain and participates in multiple neurophysiological processes. It is closely associated with the functions of neurons, synapses, and neurotransmitters (135).

### 6.4 Insulin resistance in Alzheimer’s disease animal models

In the 3xTg-AD mouse model, Alzheimer’s disease neuropathology has been shown to impair insulin signaling pathways at the blood-brain barrier (BBB), specifically manifested as diminished activation of vascular insulin receptors (136). Further studies indicate that in this model, central nervous system insulin resistance emerges earlier and progresses more rapidly than peripheral insulin resistance (137). Notably, dietary restriction interventions can improve recognition deficits in 3xTg-AD mice. The underlying mechanism may involve reduced insulin secretion, which subsequently activates GSK-3β, thereby promoting hippocampal neuronal differentiation and maturation, ultimately contributing to the recovery of learning and memory functions (138). To more directly investigate the causal relationship between insulin resistance and AD, researchers developed the APP/IR-dKI double knockout mouse model through hybridization techniques. This model exhibits systemic insulin resistance without persistent hyperglycemia. Studies found that APP/IR-dKI mice display premature onset of cognitive dysfunction, providing compelling evidence for the hypothesis that “insulin resistance promotes cognitive impairment” (139). Additionally, in APP/PS1 transgenic mice, high-fat diets significantly accelerate cognitive deficits and AD-related pathology progression by inducing obesity and insulin resistance (140). Similarly, an insulin-resistant state has been observed in the brains of 5xFAD transgenic mice, with hippocampal levels of phosphorylated PI3K and phosphorylated AKT significantly lower than in wild-type controls (141). In chemically induced models, AD models established by intracerebroventricular injection of low-dose streptozotocin show significantly reduced expression of phosphorylated insulin receptors and phosphorylated Akt in the brain. Intranasal insulin therapy effectively ameliorates this insulin signaling impairment and associated cognitive deficits (142).

In summary, extensive animal model studies consistently demonstrate that Alzheimer’s disease neuropathology is frequently accompanied by brain insulin resistance, with substantial evidence supporting this association (143). However, despite this compelling evidence, establishing a definitive causal relationship between brain insulin resistance and AD neuropathology in animal models remains a formidable scientific challenge.

### 6.5 Regulation of insulin metabolism

The half-life of insulin in the human body is extremely short, only about 4–6 minutes, and its metabolic homeostasis is mainly regulated by the insulin-degrading enzyme (IDE) (144). Notably, amyloid-beta (Aβ) and insulin are both substrates of IDE, and when IDE dysfunction occurs, it not only leads to hyperinsulinemia and glucose intolerance but also triggers an abnormal accumulation of endogenous Aβ in the brain (145), which in turn affects normal neurological function.

### 6.6 Cell-type-specific effects of insulin resistance in the brain

#### 6.6.1 Neurons

Insulin resistance leads to a significantly reduced responsiveness of neurons to insulin, manifested as mitochondrial dysfunction, decreased glucose metabolism, and elevated lactate levels. This metabolic abnormality prompts neurons to shift toward glycolysis for energy production, resulting in compensatory metabolic reprogramming. In the pathological progression of Alzheimer’s disease (AD), insulin resistance not only impairs neuronal insulin signaling but also exacerbates metabolic disturbances, thereby accelerating disease advancement (146). *In vitro* experiments further demonstrate that insulin resistance markedly compromises neuronal metabolic efficiency, leading to increased oxidative stress, reduced neuronal activity, and a decline in dopaminergic neuron count (147). Additionally, insulin resistance induces synaptic insulin resistance through a ubiquitination-dependent mechanism of synaptic protein degradation, which in turn impairs synaptic plasticity and cognitive function (148).

#### 6.6.2 Microglia

Insulin resistance impairs the metabolic homeostasis and immunoregulatory functions of microglia. Studies have demonstrated that insulin signaling in microglia is essential for maintaining metabolic balance and immune regulation, whereas insulin resistance disrupts these critical functions, leading to reduced Aβ clearance and enhanced neuroinflammation. Additionally, insulin resistance induces metabolic reprogramming in microglia, characterized by increased glycolysis (149). In high-fat diet (HFD)-induced models of insulin resistance, microglial autophagy is significantly suppressed, indicating that insulin resistance exacerbates neurodegenerative pathology by interfering with cellular clearance mechanisms (150). Collectively, these findings reveal that insulin resistance affects microglial function through multiple pathways, thereby playing a pivotal role in the pathogenesis of Alzheimer’s disease.

#### 6.6.3 Astrocytes

Astrocytes are essential for brain energy metabolism and exhibit a loss of homeostatic functions in Alzheimer’s disease (AD), which may contribute to neurodegeneration. Insulin resistance leads to impaired glucose uptake and reduced glycogen synthesis in astrocytes, thereby disrupting brain energy homeostasis (119). Furthermore, insulin resistance interferes with mitochondrial metabolism in astrocytes, manifested as abnormal mitochondrial responses to glucose. This dysfunction may result in neurovascular coupling impairment, further compromising the coordination between cerebral blood flow and glucose metabolism (151).

#### 6.6.4 Endothelial cells

Endothelial cells in the brain are critical components of the blood-brain barrier (BBB), playing a central role in maintaining brain microenvironmental homeostasis, regulating substance transport, and controlling cerebral blood flow (152, 153). Damage to endothelial cells results in the loss of tight junctions and increased barrier permeability, leading to insufficient cerebral blood perfusion, microhemorrhages, and infiltration of neurotoxic substances (154). Such damage can manifest as early as the initial stages of Alzheimer’s disease (AD) (155). Insulin resistance affects the unique properties of brain endothelial cells, rendering them more susceptible to oxidative stress. While moderate oxidative stress is necessary for insulin signaling, excessive oxidative stress exerts detrimental effects (156).

In summary, insulin resistance comprehensively impairs brain cell function through the synergistic actions of multiple cell types and pathways—including metabolic reprogramming, immune dysregulation, barrier disruption, and proteostasis imbalance—thereby establishing a core pathological foundation for AD pathogenesis. Targeting cell-specific insulin signaling pathways may represent a key strategy for intervening in AD progression.

### 6.7 Possible mechanisms by which insulin resistance affects Alzheimer’s disease

#### 6.7.1 Increase in pathologic products

Abnormal aggregation of Aβ and Tau proteins has become a typical pathological hallmark of AD (157–159). Insulin plays a key role in regulating amyloid precursor protein (APP) metabolism by promoting its degradation by the non-amyloid pathway, a process that involves both Gsk-3β-dependent and non-dependent mechanisms (160). However, IR is closely associated with amyloidosis and aberrant phosphorylation of Tau proteins in rodent and human brains (161). Research suggests a substantial association between heightened insulin resistance levels and augmented Pittsburgh compound B (PiB)uptake in the frontal and temporal regions, reflecting an increase in amyloid deposition (162). Further evidence suggests that IR may precede the onset of Aβ lesions, and a 15-year-long follow-up study revealed a significant association between midlife IR and increased cerebral amyloid load in later life (20). Furthermore, IR is not only associated with cognitive decline but also positively correlated with the accumulation of Tau biomarkers in cerebrospinal fluid (CSF) (163). Mechanistically, insulin affects Tau protein metabolism by regulating GSK3β activity, which in turn affects Tau protein levels (164). Notably, in IR states, peripheral hyperinsulinemia significantly inhibits insulin-degrading enzyme (IDE) activity, leading to an abnormal accumulation of Aβ oligomers in the brain, which further exacerbates the pathological process of AD (165).

#### 6.7.2 Blood-brain barrier dysfunction

Blood-brain barrier (BBB) dysfunction is increasingly recognized as a critical factor in the pathophysiology of Alzheimer’s disease (AD), particularly during the early stages of the disease (166, 167). Insulin resistance, which is prevalent in AD patients, is closely associated with impaired BBB integrity. Studies have demonstrated a synergistic interaction between insulin resistance and APOE genotype, jointly influencing BBB permeability, which may represent a key component in AD pathogenesis (168). Conversely, functional deficits in insulin receptors (INSR) at the BBB are correlated with amyloid-β pathology, significantly contributing to cerebral insulin resistance in AD (136). These findings suggest that BBB dysfunction not only serves as an early biomarker of AD but may also play a pivotal role in disease progression by disrupting cerebral insulin signaling and amyloid-β metabolism.

#### 6.7.3 Synaptic plasticity impairment

IR causes significant damage to synaptic plasticity and integrity. Obust evidence supporting this notion is furnished by the research conducted by Claudia A Grillo and colleagues, who adeptly constructed a model of hippocampus-specific insulin resistance. Their model efficiently attenuated insulin receptor expression within the rat hippocampus, leading to a notable decline in hippocampal neuroplasticity (169). In addition, it has been shown that insulin receptor signaling plays a key role in maintaining synaptic density, and once this signaling is impaired, it leads to the loss of synapses, a phenomenon that fully illustrates the crucial role of insulin in synapse development and maintenance (170).

#### 6.7.4 Neuroinflammation

The persistence of neuroinflammation, one of the key pathological mechanisms of AD, has been shown to lead to neuronal damage, and synaptic dysfunction, and further exacerbate pathological changes in the brain (171, 172). In-depth studies revealed a substantial direct association between microglial activation and AD-related pathological products, a finding that clarifies the strong link between neuroinflammation and the severity of AD (173). By simulating the state of hyperinsulinemia through *in vitro* experiments, researchers treated primary cultured microglia and BV2 cell lines with insulin and found that hyperinsulinemia not only stimulated microglia proliferation and drove them to M1-type polarization by promoting the production of pro-inflammatory factors, but also caused significant impairment of the membrane translocation function of GLUT4 (174). In addition, aberrant activation of neuroinflammation in the context of IR has been shown to further accelerate the progression of AD (175).

#### 6.7.5 Renin-angiotensin-aldosterone activation

IR activates RAAS, and excessive activity of this system in the brain is closely linked to the initiation and advancement of AD (176, 177). Studies have shown that angiotensin-converting enzyme (ACE) levels in cerebrospinal fluid are elevated among individuals affected by AD, a finding that further supports the important role of RAAS in AD pathology (178). In addition, patients taking angiotensin receptor blockers (ARBs) showed age-related reductions in cerebrospinal fluid Aβ levels, suggesting a potential role for ARBs in modulating markers of AD-related pathology (179). Strikingly, however, an independent study drew conclusions that differed significantly from the previous findings, demonstrating that cerebrospinal fluid and serum protein levels and activity of ACE tended to be reduced in patients with AD compared to controls (180). This paradoxical result not only reveals the complexity and diversity of the RAAS in the pathomechanism of AD but also implies the necessity of in-depth investigation of the mechanism of action of RAAS in AD and its potential therapeutic targets.

#### 6.7.6 Oxidative stress

In the pathological context of IR, the levels of oxidative damage markers within the cerebral cortex are significantly elevated, a change that reveals an enhanced oxidative stress response (181). Of particular interest is the fact that the brain exhibits an extremely high sensitivity to free radical attacks compared to other body organs (182). Accordingly, the lesions presented in the brains of individuals with AD, such as DNA damage, protein oxidation, lipid peroxidation, and accumulation of advanced glycosylation end products, are closely related to free radical attack (183). In addition, antioxidants exhibit potential value in the treatment of AD by effectively reducing the generation of reactive oxygen species (184, 185).

Although significant progress has been made in elucidating individual pathological mechanisms in Alzheimer’s disease (AD), these studies often fail to fully account for the complexity and heterogeneity of its clinical manifestations, highlighting the limitations of a single-target approach.

For example, the correlation between amyloid-β (Aβ) pathology and cognitive decline is not always direct; some Aβ-positive individuals remain cognitively normal for extended periods, suggesting that Aβ deposition alone is insufficient to explain clinical heterogeneity (186). Moreover, therapeutic strategies targeting Aβ or tau protein in isolation have not yielded ideal outcomes in clinical trials (187, 188). Neuroinflammation is widely recognized as a key pathological feature of AD, yet its precise role remains incompletely understood. In particular, whether neuroinflammation acts as a primary driver or a secondary phenomenon in AD pathogenesis continues to be debated (189). Neuroinflammation exhibits a “double-edged sword” characteristic in AD: while moderate inflammatory responses may be protective, excessive suppression could be detrimental. For instance, microglial activation not only aids in Aβ clearance but may also exacerbate neuronal damage (190). The association between the renin-angiotensin-aldosterone system (RAAS) and AD pathology has been frequently proposed, but its underlying molecular mechanisms are not yet fully clarified. Some studies suggest that RAAS-targeting drugs (e.g., ARBs) may reduce AD risk (191, 192), while others report inconsistent findings. For example, although a clinical trial of losartan demonstrated reduced brain volume loss, it did not conclusively prove cognitive benefits (193). Furthermore, while oxidative damage is closely associated with Alzheimer’s disease (AD), antioxidant therapies have failed to achieve significant clinical efficacy, reflecting the complexity of oxidative stress in AD pathogenesis (194). Moreover, the causal relationships between oxidative stress and other pathological features—such as amyloid-β (Aβ) deposition and tau hyperphosphorylation—remain controversial. It remains challenging to determine whether oxidative stress acts as a primary event or a secondary phenomenon in the disease process.

Consequently, as noted by other researchers, AD remains a poorly understood disease with a complex, multifactorial pathogenesis (195). In this context, insulin resistance offers a theoretical bridge that closely links multiple mechanisms, including neuroinflammation, oxidative stress, and RAAS activation. Adopting this integrative perspective may deepen our understanding of AD pathophysiology, provide a more comprehensive view of the disease, and ultimately inform the development of more effective prevention and treatment strategies.

## 7 Potential therapeutic strategies

### 7.1 Pharmacologic interventions for insulin resistance

#### 7.1.1 GLP-1 receptor agonists

Glucagon-like peptide-1 (GLP-1) receptor agonists are standard therapies for type 2 diabetes and obesity, lowering blood glucose and body weight by promoting insulin secretion, inhibiting glucagon release, and inducing satiety via central nervous system actions (196). In neuroprotection, liraglutide (a GLP-1 receptor agonist) shows therapeutic potential. Six months of treatment significantly enhances blood-brain barrier glucose transport, improves cerebral glucose metabolism, and reverses AD-related brain glucose transport abnormalities, supporting its role in brain energy metabolism and AD therapy (197). Additionally, 12 weeks of treatment improves brain connectivity in high-risk AD individuals (198). However, a systematic review notes that while GLP-1 receptor agonists offer metabolic and neuroprotective benefits, they do not significantly alter amyloid-β or tau biomarkers or improve cognitive function (199). Overall, liraglutide holds promise for cognitive function and neuroprotection, but its definitive efficacy and mechanisms require further validation through high-quality studies.

#### 7.1.2 Intranasal insulin

Intranasal insulin administration is a non-invasive delivery method that directly transports insulin to the brain via the nasal mucosa, effectively bypassing the blood-brain barrier. This approach has shown potential value in treating neurological disorders such as Alzheimer’s disease (AD). One study reported that, compared to the placebo group, patients receiving conventional insulin therapy exhibited significant memory improvements at 2 and 4 months, with statistically significant differences (p < 0.03) (200). However, not all studies have observed similar cognitive benefits. For instance, a 12-month randomized controlled trial (RCT) demonstrated that intranasal insulin treatment did not lead to significant cognitive improvements in patients with mild cognitive impairment (MCI) or Alzheimer’s disease (201). Furthermore, a systematic review of seven RCTs involving patients with AD or MCI indicated that intranasal insulin therapy only modestly improved story recall performance in APOE4-negative (APOE4[-]) individuals, while its effects on other cognitive domains remained limited (202).

#### 7.1.3 Metformin

Metformin, a drug widely used in the antidiabetic field, has received much attention in recent years for its potential role in the therapy of AD. A meta-analysis showed that long-term and high-dose use of metformin was significantly associated with a reduced risk of developing AD in elderly diabetic patients (203). It should be noted, however, that the results of several studies have shown that metformin treatment did not result in the expected significant improvement in patients’ cognitive performance, and some studies have suggested that it may increase the risk of developing AD (204–206). In particular, a meta-analysis of 10 studies found that metformin treatment may adversely affect the risk of developing AD in Asian populations (206). These conflicting findings suggest that the administration of metformin for AD should be evaluated more cautiously, taking into account the potential differences in different populations and the heterogeneity of their responses to the drug.

#### 7.1.4 PPAR-γ agonists

Activation of PPAR-γ receptors plays multiple roles in regulating a wide range of biological processes, including lipid metabolism, inflammatory responses, and neuroprotection (207). Studies have shown that PPAR-γ agonists significantly improve spatial learning and memory in animal models, demonstrating their great potential in the therapeutic field of AD (208, 209). In particular, pioglitazone, a widely used PPAR-γ agonist, has shown potential benefits for AD patients in systematic evaluations, although these preliminary findings require further validation to establish their value for clinical application (210).

### 7.2 Lifestyle interventions

Studies have shown that limiting net carbohydrate intake to 130 grams per day may be effective in slowing the rate of cortical atrophy and contribute to lower insulin levels (211). Furthermore, in overweight and obese populations, regular exercise has shown significant effects in restoring insulin sensitivity in the brain (212). More importantly, comprehensive lifestyle Interventions markedly improve cognitive function in individuals with MCI or early AD, and may slow disease progression by improving IR and lowering insulin levels (213).

## 8 Conclusion

**Figure 2:**
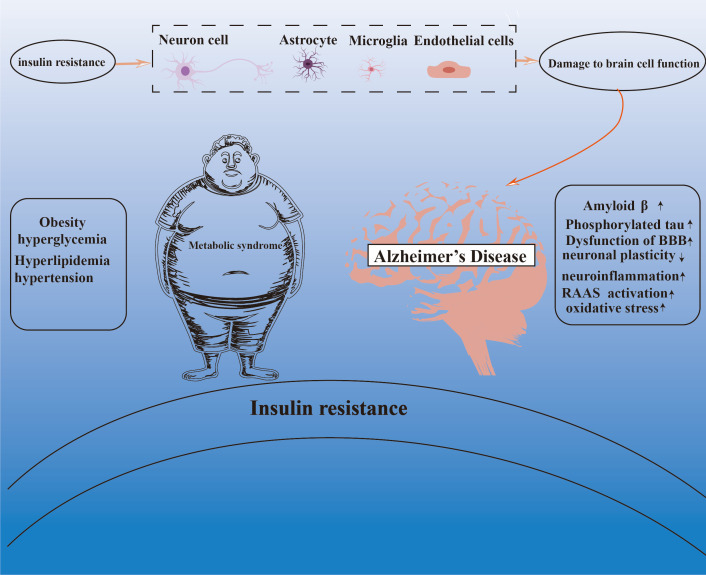
The central role of insulin resistance in the Mets-AD connection.

This review focuses on the central mechanism of IR in the association between MetS and AD. By systematically combing and analyzing the research findings of recent years, we demonstrate that IR plays a key bridging role between MetS and AD (**Figure 2**). On the one hand, there is a close association between IR and the components of the MetS; on the other hand, IR is a key factor in the pathogenesis of AD. However, current research exhibits significant limitations in elucidating this complex association. For instance, most studies overlook the impact of sex differences on the pathogenesis of metabolic syndrome (MetS) and Alzheimer’s disease (AD). As documented in the literature, women with MetS are more susceptible to AD than men (18), a disparity potentially amplified by insulin resistance-related MetS, as supported by previous findings (214, 215). Additionally, existing studies often treat mechanisms such as neuroinflammation, oxidative stress, overactivation of the renin-angiotensin-aldosterone system (RAAS), and mitochondrial dysfunction as independent factors, lacking in-depth critical analysis and cross-mechanistic integration. This “mechanism-listing” approach fails to adequately elucidate how these pathways dynamically interact, mutually amplify, and collectively drive neurodegeneration. For example, insulin resistance can directly induce central neuroinflammation, while inflammatory cytokines (e.g., TNF-α, IL-1β) released by activated glial cells further exacerbate peripheral and central insulin signaling impairment, creating a vicious cycle. The existence of such “crosstalk” and “positive feedback loops” among mechanisms implies that therapeutic strategies targeting a single pathway may yield limited efficacy or even be counteracted by compensatory activation of other pathways. Nevertheless, it is undeniable that the importance of IR in the pathogenesis of AD should not be underestimated, and considering it as a promising candidate for the intervention and management of AD holds great and far-reaching significance. Therefore, future studies should focus on the development of multidimensional intervention strategies that focus on the optimization of IR, to deeply analyze the complex pathological process of AD and further promote its application and development in the treatment of neurodegenerative diseases.
